# Suture-Button Versus Syndesmotic Screw Fixation of Ankle Fractures: A Comparative Retrospective Review Over One Year

**DOI:** 10.7759/cureus.17826

**Published:** 2021-09-08

**Authors:** Bakhat Yawar, Brian Hanratty, Ayeisha Asim, Aamir K Niazi, Abdul M Khan

**Affiliations:** 1 General Surgery, The Western Trust HSCNI, Derry/Londonderry, GBR; 2 Trauma and Orthopaedics, Altnagelvin Area Hospital, Derry/Londonderry, GBR; 3 Geriatrics, Altnagelvin Area Hospital, Derry/Londonderry, GBR; 4 Trauma and Orthopaedics, Medway Maritime Hospital, Gillingham, GBR; 5 Orthopaedics, Airedale Hospital Trust, Steeton, GBR

**Keywords:** tightrope, syndesmotic injury, ankle fracture, syndesmotic screws, trauma

## Abstract

Background

Syndesmotic fixation is essential in the management of unstable ankle fractures with syndesmotic disruption. It can be achieved either by rigid fixation with screws or dynamic fixation with suture-button devices. Syndesmosis screws are considered the gold standard for the stabilisation and fixation of distal tibiofibular syndesmotic injuries. We use both types of devices in our unit for the stabilisation of syndesmotic injuries. In our department, Arthrex TightRope® (Arthrex, Naples, FL) is the preferred suture-button device for the dynamic fixation of distal tibiofibular syndesmosis. Some studies have reported better outcomes with the use of suture-button devices. In this study, we aim to compare the outcomes with the use of both these devices.

Methodology

This retrospective cohort study was conducted from 1 June 2019 until 31 May 2020 which included all patients who underwent syndesmotic fixation. Data were collected from the Northern Ireland Electronic Care Record and the Northern Ireland Picture Archiving and Communication System. Primary outcomes included reoperation rates due to failure of syndesmotic hardware. Secondary outcomes included the comparison of complications (e.g. infection, wound problems, or loss of reduction) radiographic fixation/stability, duration of follow-up, and significant ongoing symptoms at the time of discharge from the clinic.

Results

A total of 53 patients underwent syndesmotic stabilisation during this period. In total, 34 patients underwent fixation with TightRope and 19 underwent syndesmotic screw fixation. Three patients (9%) had reoperation due to any cause in the TightRope group compared to four patients (21%) in the syndesmotic screw group. All four patients in the syndesmotic screws group underwent implant removal due to failure or symptoms due to implant. Only one patient (3%) in the TightRope group underwent implant removal due to ongoing pain [the other two (6%) patients included one with lateral ankle abscess and one with long distal fibula screws, both unrelated to the use of TightRope and TightRope left in situ at reoperation]. Rediastasis was reported in one (5.2%) patient with syndesmotic screws. The median duration of follow-up was 82 days in the syndesmotic screw group compared to 77.5 days in the TightRope group.

Conclusions

Based on our data, we report a lower incidence of implant-related problems and the need for implant removal with the use of TightRope. Quicker return to weight-bearing and slightly better radiographic stabilisation were noted as well. Our study has its limitations in terms of a small number of patients and the absence of functional outcomes as these were not routinely recorded at the time of discharge from the clinic.

## Introduction

A syndesmosis is a joint comprising two bones linked by a fibrous membrane. The distal tibiofibular joint is an example of a syndesmotic joint, which comprises four ligaments, the anterior inferior tibiofibular ligament, the posterior inferior tibiofibular ligament, the transverse ligament, and the interosseous membrane [1]. The syndesmotic ligament along with the collateral ligament maintains stabilisation and congruence of the talocrural joint.

Ankle fracture is one of the most common fractures with an overall incidence of approximately 75 per 100,000 individuals [2]. Incidence of syndesmotic injury has been described as high as 55% in Weber B-type fractures and 100% in Weber C-type fractures of the ankle. Instability has been reported as high as 45% in Weber B-type fractures and 70% in Weber C-type fractures [3]. Persistent ankle pain, poor functional outcomes, and early osteoarthritis are potential complications of misdiagnosed or inadequately treated syndesmotic injuries. Therefore, it is important to maintain syndesmotic reduction after such injuries [4]. The aim is to achieve anatomical reduction of the ankle joint which is described as the most important predictor of good clinical outcomes [5].

Traditionally, syndesmotic screws were considered the gold standard for treating syndesmotic injuries, but more recently, suture-button devices such as Arthrex TightRope® (Arthrex, Naples, FL) are becoming increasingly popular in managing such injuries [4]. Previous studies have shown advantages of suture-button devices over syndesmotic screws such as allowing physiological movement at the syndesmosis, lower incidence of rediastasis, lower incidence of implant removal, quicker rehabilitation, better quality of life, and cost-effectiveness [4,6-9].

In our Trauma and Orthopaedics department at Altnagelvin Area Hospital, we received feedback from our consultants and senior registrars regarding a series of complications over a course of two months related to fixation of ankle syndesmotic injuries with a suture-button device. Therefore, in this study, we aimed to investigate these concerns. We decided to study the results of both suture-button and syndesmotic screw fixation in ankle fractures over a period of one year from June 2019 till May 2020 which also provided us with a one-year follow-up interval to identify complications with the use of both these devices.

## Materials and methods

Study design

This was a retrospective cohort study that involved collecting data regarding ankle fractures with a syndesmotic injury who underwent operative fixation and stabilisation with either a syndesmotic screw or a suture-button device (TightRope).

Inclusion and exclusion criteria

We included all patients presenting with ankle fractures and distal tibiofibular syndesmotic injury diagnosed either radiographically or intra-operatively (intra-operative diagnosis was based on stress testing the syndesmosis under direct or radiographic guidance). Ankle fractures without syndesmotic injury, pilon fractures of the distal tibia, and open ankle fractures were excluded from the study.

Participating centres

Patients who presented to Altnagelvin Area Hospital or the emergency departments of peripheral hospitals served by our hospital (namely, South West Acute Hospital, Causeway Hospital, and Omagh Hospital) with ankle fractures and underwent syndesmotic fixation between 1 June 2019 and 31 May 2020 under the Orthopaedics team in Altnagelvin Area Hospital were included in the study.

Data collection

Patients were identified from Orthopaedics trauma theatre lists and data were collected from the Northern Ireland Electronic Care Record. No patient identifiable data were collected. The Northern Ireland Picture Archiving and Management System was used to review X-ray images and perform measurements.

Outcomes

The primary aim of our study was to compare the reoperation rates in patients managed with syndesmotic screws and suture-button devices. Secondarily, we compared the incidence and causes of complications, time to weight-bearing, and post-operative radiological analysis [i.e. risk of malreduction based on medial clear space (normal <4 mm) and tibiofibular overlap (normal >6 mm)]. Baseline characteristics of both groups were also identified.

Data analysis

Reoperation rates in general for both groups as well as reoperation rates due to implant failure or other implant-related complications were identified. Data on patient demographics and complications were collected with a paper data collection tool and the data were then transferred to a Microsoft Excel table. SPSS software (IBM Corp., Armonk, NY) was used to compare reoperation rates in general for both groups and reoperation rates due to implant failure or other implant-related complications using odds ratio analysis. For continuous data variables, such as duration of follow-up and time to weight-bearing, Student’s t-test was used to compare the means of both groups using SPSS.

## Results

Over the study period, 129 patients underwent operative management of their ankle fractures in our department. Among these patients, 53 had ankle fractures with associated distal tibiofibular syndesmotic injury with instability and underwent syndesmotic stabilisation with either syndesmotic screws or TightRope (type of suture-button device used in our department). TightRope was used for syndesmotic stabilisation in 34 patients, whereas syndesmotic screws were used in 19 patients. Some of the baseline patient characteristics are described in Table 1. This table also classifies the fractures based on the anatomical type or the Lauge-Hansen classification in both groups. The primary and secondary outcomes are described in Table 2.

**Table 1:** Baseline characteristics of patients included in the study. SD: standard deviation; HTN: hypertension; peripheral vascular disease

Variable	Category	TightRope patients		Syndesmotic screw patients	
		N = 34	Summary	N = 18	Summary
Age (mean ± SD)	-	34	44.59 ± 17.22	18	50.8 ± 23.8
Sex	Male	34	22 (65%)	18	7 (39%)
	Female		12 (35%)		13 (61%)
Comorbidities	HTN	34	2 (6%)	18	3 (17%)
	Diabetes		1 (3%)		1 (6%)
	PVD		1 (3%)		-
	Hypercholesterolemia		2 (6%)		1 (6%)
Smoker	No or unsure	34	32 (94%)	18	16 (88%)
	Yes		2 (6%)		2 (12%)
Anatomical type	Weber B	34	2 (6%)	18	3 (17%)
	Weber C		2 (6%)		2 (11%)
	Bimalleolar		12 (35%)		6 (33%)
	Trimalleolar		13 (38 %)		7 (39%)
	Maisonneuve		5 (15%)		-
Lauge-Hansen classification	SER4	34	14 (41%)	18	8 (42%)
	PER4		15 (44%)		11 (58%)
	Maisonneuve		5 (15%)		-

**Table 2:** Comparison of outcomes with TightRope and syndesmotic screws. OR: odds ratio; SD: standard deviation

Outcome	Category	Patients		OR (T/S) or mean ± SD (mm)	P-value
		N	n (%)		
Reoperation due to any cause	TightRope	34	3 (11.63%)	OR = 0.34 (0.07–1.72)	0.09
	Syndesmotic screw	18	4 (22%)	-	-
Reoperation due to implant in question	TightRope	34	1 (3%)	OR = 0.11 (0.011–1.036)	0.026
	Syndesmotic screw	18	4 (22%)	-	-
Medial clear space (mm)	TightRope	34	-	Mean = 3.18 ± 0.85	0.19
	Syndesmotic screw	18	-	Mean = 3.57 ± 1.85	-
Tibiofibular overlap (mm)	TightRope	34	-	Mean = 7.68 ± 1.80	0.21
	Syndesmotic screw	18	-	Mean = 3.57 ± 1.85	-
Mean time to weight-bearing (weeks)	TightRope	34	-	OR = 1.23	0.01
	Syndesmotic screw	18	-	OR = 3.15	-

Primary outcomes

The primary outcomes were to measure the incidence of reoperation due to any cause and the incidence of reoperation specifically due to problems with the implant used for syndesmotic fixation. Among the 19 patients who underwent syndesmotic screw insertion, four (21%) had a reoperation. Among the patients who had their syndesmosis fixed with screws, all underwent reoperation due to problems with the implant. One patient had rediastasis. This patient originally had a complex injury to the ankle which was managed with retrograde fibula nail, lag screws to fix medial malleolus, and syndesmotic screw insertion. Three months later the patient experienced ankle pain and instability, and X-rays showed rediastasis. She subsequently underwent ankle arthrodesis with distal fibulectomy. Two patients in this group had syndesmotic screws removed due to ongoing pain attributed to the screws, and one patient underwent screw removal due to screw backout. Among the 34 patients with TightRope fixation of the syndesmosis, only three (9%) underwent reoperation. One patient had the TightRope device removed as the patient had ongoing anteromedial pain attributable to the TightRope. One patient had washout for a lateral wound infection which developed into an abscess along the lateral incision site, not attributed to the device. One patient had long intra-articular distal fibula screws which led to ankle pain. This patient was planned to only have these screws removed and exchanged as there was no problem due to the TightRope device.

Secondary outcomes

In the group of patients managed with TightRope, a total of six patients had complications. In addition to the complications mentioned for TightRope in the primary outcomes section, one patient had a stitch abscess which settled with antibiotics, one had wound ooze for approximately three months (this patient returned to work early despite being warned against it and was walking up to 20,000 steps/day in the initial two to three weeks). One patient had a pulmonary embolism. For syndesmotic screw patients, the complications are as described in the primary outcomes section above. Radiological fixation was noted to be better in the TightRope group but was not statistically significant. Time to weight-bearing was decided by consultants performing the surgery. Among the patients managed with TightRope, five had significant stiffness of ankle joint at the last clinic follow-up and three had significant pain which was affecting their activities of daily living. Among the patients managed with syndesmotic screws, one patient had significant stiffness and two had significant pain.

## Discussion

Ankle fracture is the most common fracture sustained in patients 20 to 65 years of age, and syndesmotic injury is reported in 13% of all patients with ankle fractures and 20% of ankle fractures requiring internal fixation [10]. Up to 0.5% of ankle sprains without fracture are reported to have syndesmotic disruption [10,11]. Untreated syndesmotic injuries lead to pain and instability of the ankle joint. Treatment of syndesmotic injuries with static or dynamic fixation remains a hotly debated topic in orthopaedics. Although syndesmotic screws have been described as the gold standard for managing syndesmotic injuries for a long time, with the advent of suture-button devices such as Arthrex TightRope, the interest has been gradually increasing in dynamic fixation of syndesmosis injuries over the last 10-15 years [4]. Suture-button devices such as tightropes are said to allow physiological movement at the distal tibiofibular syndesmosis. The dynamic role of the fibula was described by Scranton et al. in 1976 in maintaining ankle mortise stability during gait [12].

The use of syndesmotic screws has been described to lead to complications such as synostosis, screw breakage, or recurrent diastasis [13,14], even though the risk of these complications is described to be relatively low. Complications are more commonly seen in Weber C-type fractures after syndesmotic screw stabilisation rather than Weber B-type fractures [14]. In addition, removal of syndesmotic screws was previously a common practice, but studies have suggested complications with screw removal such as significant wound infection after screw removal, recurrent diastasis, and unnecessary removal of the broken screw [15]. Andersen et al. went as far as to recommend prophylactic antibiotics in elective syndesmotic screw removal [16]. Therefore, some studies recommend screw removal in select patients with ongoing symptoms attributable to the screws [14-16], which is the current practice in our department as well. Tucker et al. described good functional outcomes with syndesmotic screw fixation where screws were left in situ with a follow-up period of 31 months [17].

Arthrex TightRope is a type of suture-button device used for syndesmotic stabilisation which is a non-absorbable fibrewire loop placed across the distal tibiofibular syndesmosis and tightened through metal cortical endobuttons. Storey et al. described a few complications of the use of TightRope such as osteomyelitis, aseptic osteolysis, and rediastasis due to failure of implant insertion technique in 2012. They suggested some modifications to the insertion of the device including a medial incision to ensure the endobutton sits on the tibial cortex and insertion through the fibular plate or a small plate to act as a washer to prevent the lateral button pulling through the thin fibular cortex [18]. Other complications reported include pain, discomfort, and syndesmosis ossification [4]. Recently, Chen et al. reported in 2020 that reduction accuracy should be kept in mind while using tightropes in Weber C-type fractures, and patients should be monitored closely for rediastasis in these types of injuries [19]. Laflamme et al. reported in 2015 that dynamic fixation devices provide better functional and radiographic outcomes compared to static fixation devices [7]. However, Kortekangas et al. [6] reported no significant difference in functional outcomes between patients treated with syndesmotic screws or TightRope. Several studies have reported a lower risk of malreduction with the use of TightRope [5,7,20-22]. A systematic review by Zhang et al. [4] concluded similar functional outcomes and post-operative complication rates in both groups but reported suture-button fixation may lead to a better range of movement, earlier rehabilitation, lower rate of implant removal, implant failure, and lower risk of malreduction in patients managed with suture-button devices; however, they noted publication bias in the studies included. Another systematic review by McKenzie et al. revealed similar findings as well as lower reoperation rates in patients treated with tightropes [23].

Our study was limited due to its small sample size and lack of data on functional outcomes. However, we did note that reoperation rates were lower in patients treated with TightRope (especially accounting for reoperation rates due to implant problems).

The time at which patients were allowed to weight-bear was consultant-specific in our department. There is no protocol in our department regarding time to allow weight-bearing in the setting of both screw and suture-button fixation to date. In the syndesmotic screw, the mean time to weight-bearing was 3.15 weeks, and only eight patients (42%) were allowed to weight-bear at zero weeks in lightweight compression system dressing (AndoFlex dressing) early after surgery. Furthermore, nine (46%) patients were allowed to weight-bear only at six weeks after a long period of being protected as non-weight-bearing in a cast or a walking boot. In the TightRope group, 25 (74%) patients were allowed to weight-bear at zero weeks and the mean time to weight-bearing was 1.23 weeks. It is important to mention here that surgeons may allow patients treated with TightRope earlier weight-bearing as syndesmotic screws are at risk of breaking with early mobilisation. Some consultants prefer to weight-bear patients on day one in protective dressing or boot, whereas some consultants prefer to keep patients non-weight-bearing for a few weeks (up to six weeks) after operative fixation in our department.

It is noteworthy to mention that none of the consultants in our department removes syndesmotic screws on an elective list as a planned procedure after an interval, and these screws are only removed in our department in case of complications. We note that rigid stabilisation was preferred in some of the more complex ankle fractures and provided good results. We recommend that TightRope and syndesmotic screws both provide good outcomes, although TightRope may be slightly superior in lowering reoperation rates. Further studies such as a randomised clinical trial currently being organised by Doll et al. [24] will likely shed more light on this issue and further clarify the merits and demerits of both treatment options. With the review of our results, we shall continue to recommend both treatment modalities according to the patient’s surgeon preference and clinical needs.

## Conclusions

In our department, the results of this study provided reassurance that suture-button devices remain a safe surgical option for the management of ankle fractures with syndesmotic injuries, especially in light of the recent series of problems noticed in our clinics. This has restored confidence in the use of this relatively new device among our consultants. Recent literature also endorses the safety and efficacy of suture-button devices. Further prospective studies and randomised trials are needed to clarify the advantages and disadvantages of both treatment options.
